# Genome-Wide Analysis of Transcription Factor *R2R3-MYB* Gene Family and Gene Expression Profiles during Anthocyanin Synthesis in Common Walnut (*Juglans regia* L.)

**DOI:** 10.3390/genes15050587

**Published:** 2024-05-05

**Authors:** Dongjun Zuo, Yujie Yan, Jiayu Ma, Peng Zhao

**Affiliations:** Key Laboratory of Resource Biology and Biotechnology in Western China, Ministry of Education, College of Life Sciences, Northwest University, Xi’an 710069, China; zack39723@gmail.com (D.Z.); yujieyan0103@163.com (Y.Y.); majiayu@stumail.nwu.edu.cn (J.M.)

**Keywords:** *Juglans*, *JrR2R3-MYB*, transcriptome anthocyanin biosynthesis

## Abstract

The *R2R3-MYB* gene family, encoding plant transcriptional regulators, participates in many metabolic pathways of plant physiology and development, including flavonoid metabolism and anthocyanin synthesis. This study proceeded as follows: the *JrR2R3-MYB* gene family was analyzed genome-wide, and the family members were identified and characterized using the high-quality walnut reference genome “Chandler 2.0”. All 204 *JrR2R3-MYBs* were established and categorized into 30 subgroups via phylogenetic analysis. *JrR2R3-MYBs* were unevenly distributed over 16 chromosomes. Most *JrR2R3-MYBs* had similar structures and conservative motifs. The *cis*-acting elements exhibit multiple functions of *JrR2R3-MYBs* such as light response, metabolite response, and stress response. We found that the expansion of *JrR2R3-MYBs* was mainly caused by WGD or segmental duplication events. Ka/Ks analysis indicated that these genes were in a state of negative purifying selection. Transcriptome results suggested that *JrR2R3-MYBs* were widely entangled in the process of walnut organ development and differentially expressed in different colored varieties of walnuts. Subsequently, we identified 17 differentially expressed *JrR2R3-MYBs*, 9 of which may regulate anthocyanin biosynthesis based on the results of a phylogenetic analysis. These genes were present in greater expression levels in ‘Zijing’ leaves than in ‘Lvling’ leaves, as revealed by the results of qRT-PCR experiments. These results contributed to the elucidation of the functions of *JrR2R3-MYBs* in walnut coloration. Collectively, this work provides a foundation for exploring the functional characteristics of the *JrR2R3-MYBs* in walnuts and improving the nutritional value and appearance quality of walnuts.

## 1. Introduction

The common walnut (*Juglans regia* L.) is one of the most important woody plant resources in the world [1]. The economic value of walnut plants is reflected in their nutrient-rich nuts and high-quality timber, each of which are extremely affected by phenolic compound synthesis pathways [2,3]. There is clinical evidence that walnuts can prevent coronary heart disease and promote cardiovascular health [4], and these benefits are closely related to the fact that walnuts are abundant in polyphenols, especially flavonoids. Common walnuts have green leaves and husks and light yellow to brown seed coats [5], but the ‘Zijing’ walnut variety currently found in Beijing, China, has purplish-red branches, leaves, flowers, husks, and seed coats and is rich in anthocyanins [6]. Walnut color is one of the key attributes that promote walnut sales and cater to consumer preferences [7], and walnuts with a purplish-red seed coat are favored by consumers for their rich anthocyanin content. However, the important genes that regulate the color of walnuts are still largely unknown. Anthocyanins, which are flavonoids, are indespensible components with regard to plant coloration [8]. The accumulation of anthocyanins can allow plants to resist a variety of environmental stresses, attract pollinators, and spread fruit [9,10]. In addition, anthocyanins are anti-inflammatory, inhibit bacteria, prevent cardiovascular disease, lower blood sugar levels, improve vision, prevent Alzheimer’s disease and cancer, etc [11]. In recent years, researchers have also found that anthocyanins can alleviate psychological disorders such as depression in adolescents [12]. Therefore, the synthesis of anthocyanins is essential for plant growth and attracting consumers. Anthocyanin metabolism pathways in plants have been investigated widely [13,14]. *MYB*, *bHLH*, and *WD40* are essential transcription factors that regulate these pathways [15], and the *MYB* gene family plays the most critical role in fruit coloring among them [16]. *MdMYB10* can be combined with its own promoter to control the red coloration of apples [17], and *MdMYB110a* has also been found to synthesize anthocyanins in the cortex of red-fleshed apples in the later stages of maturity [18]. Grape *VvmybA1* and its homologs *VlmybA1-1* and *VlmybA1-2* can regulate anthocyanins in purple grapes [19]. It is worth exploring whether the *MYB* gene family also plays a critical part in walnut color regulation.

*MYB* is the largest family that plays critical parts in transcriptional regulation in plants [20]. All MYB factors are characterized by a conserved DNA-binding domain, which typically consists of 1–3 incomplete repeats (R1, R2, and R3). Every duplicate contains a helix–turn–helix motif variation, which creates a hydrophobic core in the 3D HTH architecture [21]. Moreover, each imperfect repeat consists of approximately 51 or 52 amino acids, containing three conserved tryptophans, segregated by 18 or 19 amino acid remnants [22]. According to the number of MYB domains, they can be organized into multiple subfamilies, mainly including *1R-MYB*, *R2R2-MYB*, *R1R2R3-MYB,* and *4R-MYB*. As an uncommon type, *5R-MYB* also exists in MYB gene family [23]. *R2R3-MYB* is the most numerous and powerful subtribe of the *MYB* gene family, and the genes in this subfamily all contain two repetitive domains [8,24]. The R2R3-MYB conserved domains are normally positioned at the end of a protein’s N-terminus, while the C-terminus of a protein varies considerably and often functions as a transcriptional activation or repression domain [25]. Recent research showed that *CgsMYB12* is involved in the formation of anthocyanin pigments at the base of *Clarkia gracilis* ssp. *sonomensis* petals [26]. The activator-type *R2R3-MYB* gene *PpMYB18* in *Prunus persica* entrains balanced anthocyanin and proanthocyanidin accumulation in the inhibitory-type gene [27]. *SsMYB1* can be positively regulated via anthocyanin biosynthesis by stimulating the *SsDFR1* and *SsANS* and influencing leaf discoloration in *Sapium sebiferum Roxb* [28]. It can be gleaned from the above that the *R2R3-MYB* gene family plays crucial roles in the anthocyanin synthesis pathway. So far, the *R2R3-MYB* gene family has been identified in many species, for example, maize, soybean [29,30], *Gossypium raimondii* [31], *Medicago truncatula* [32], and octoploid *Fragaria* × *ananassa* [33]. Phylogenetic trees of 126 AtR2R3-MYB proteins have been constructed in *Arabidopsis*, and 90 of them are divided into 23 subgroups (S1-S25, without S8 and S17) according to the evolutionary relationship [34]. Nevertheless, there has not been a comprehensive and systematic genome-wide analysis of the *JrR2R3-MYB* gene family, and little is known about the key *R2R3-MYB* genes in walnut coloration.

Accordingly, we conducted a genome-wide analysis of walnut; identified and named MYB members, determined chromosome locations, performed collinearity analysis, determined phylogenetic relationships and physicochemical properties, made subcellular location predictions, and ascertained the promoter characteristics, conservative motifs, gene structures, and expression profiles of *JrR2R3-MYB* members. Furthermore, 9 *JrR2R3-MYB* genes that might be involved in anthocyanin synthesis in the ‘Zijing’ walnut vairety were discovered. This study provides a foudation for an intensive study of novel *R2R3-MYB* genes in anthocyanin synthesis and will help to further uncover the functional characteristics of *JrR2R3-MYBs* in walnuts. Meanwhile, it provides important clues for improving the nutritional value and appearance quality of walnuts to attract consumers.

## 2. Materials and Methods

### 2.1. Genome-Wide Identification of JrR2R3-MYBs

To obtain *JrMYB* candidate members, 132 AtMYB sequences were obtained from TAIR [35] and employed as the query. The walnut reference genome was obtained from NCBI (Chandler 2.0) [36]. Local BlastP was used to find *JrMYB* candidate members with E-values < 1 × 10^−5^; then, the candidate members without SANT domain were removed by searching in SMART [37]; finally, all members of the *JrMYB* gene family were determined. To divide *JrMYB* genes into subfamilies, the conserved domains of all members were visualized using TBtools software [38] based on the SMART results.

### 2.2. Chromosome Location and Collinearity Analysis of JrR2R3-MYBs

The locations of all *JrMYBs* on the chromosome were displayed using TBtools software [38]. Based on the distribution information, all members of the *JrMYB* family were named. MCScanX software [39] was employed to determine the gene collinearity relationships among *JrR2R3-MYBs*, and Circos software [40] was employed for visualization. Analysis of the collinearity of the *JrR2R3-MYBs* between walnut and three other selected species (*Arabidopsis*, *J. mandshurica*, and *J. nigra*) was carried out using MCScanX software [39]. Genomic data on *Arabidopsis* were obtained from TAIR [35]. The genomes of *J. nigra* [41] and *J. mandshurica* [42] were obtained in our previous study.

### 2.3. Phylogenetic Analysis of JrR2R3-MYBs

We constructed a maximum likelihood (ML) phylogenetic tree based on the protein sequences of JrR2R3-MYBs, AtR2R3-MYBs, and OsR2R3-MYBs [43] using IQ-tree software [44] (Bootstarp:1000; Best BIC score model: JTT + R10) and beautifiedit using iTOL [45].

### 2.4. Characteristic Information regarding JrR2R3-MYB Proteins

The physicochemical properties of JrR2R3-MYBs were determined using ExPASy [46]. The prediction of subcellular location was performed using WoLFPSORT [47].

### 2.5. Conserved Motif, Gene Structure, and Cis-Element Analysis of JrR2R3-MYBs

Conserved motifs in JrR2R3-MYBs were detected with the MEME Suite [48], and the maximum number of motifs was determined to be 20. All *JrR2R3-MYBs* structures were analyzed using the GSDs [49] and visualized via TBtools software [38]. *Cis*-elements were determined by searching the sequences of the promoter region (2000 bp upstream of the translational start sites of genes) using PlantCARE [50].

### 2.6. JrR2R3-MYB Transcriptome Pattern Analysis and qRT-PCR Experiments

To perform transcriptome analysis, multi-organ gene expression data were obtained from the publicly available Sequence Read Archive database [51]. Gene expression data for red and green walnut were retrieved from NCBI (GSE162007, and PRJNA688391) [7], where leaves and peels were obtained from red (RW-1) and green (Zhonglin-1) walnuts. Then, to identify the *JrR2R3-MYBs* associated with the regulation of walnut color development, we selected the leaves of walnut varieties ‘Zijing’ and ‘Lvling’ for transcriptome sequencing. The ‘Zijing’ walnut is an entirely purplish-red tree, including branches, stems, leaves, female flowers, husks, and seed coats. ‘Zijing’ walnut is rich in anthocyanins, resulting in a purplish-red color all over its body, while ‘Lvling’ walnut is a common green variety that has green leaves, green male flowers, green husks, pale-yellow female flowers, yellow kernels, and pale-yellow inner seedcoats. This variety is characterized by large fruits, high kernel yields, and high fat and protein content. In addition, because of its good resistance, it is loved by fruit growers and widely cultivated throughout China (Figure S1) [52]. All samples were collected in May from six-year-old saplings at the Xi’an Botanical Garden in Shannxi, China. The raw data were initially filtered to extract high-quality clean data. Fitness sequences and low-quality reads were eliminated from the raw reads. Reads were mapped to the Chandler v2.0 genome using HISAT2 software [53], and then the mapped reads were arranged using StringTie [54] with default parameters. The gene expression levels of FPKM values were used to measure a gene or transcript through StringTie [55]. Differential expression analyses were processed using DESeq2 [56]. *JrR2R3-MYBs* were screened from the differentially expressed genes (DEGs) of the transcriptome, and we investigated whether they were associated with the regulation of anthocyanin synthesis based on FPKM values. To screen for genes involved in walnut coloration, we constructed a phylogenetic tree (ML; Bootstarp:1000) showing the different *JrR2R3-MYBs* and other *R2R3-MYBs* known to participate in anthocyanin synthesis, such as *ZmC1*, *AtMYB123*, *MdMYB10*, *FaMYB10*, and *ROSEA1*.

To inquire into the expression patterns of anthocyanin synthesis associated with *JrR2R3-MYBs* in walnuts, we collected the leaves of the ‘Lvling’ and ‘Zijing’ walnut varieties at the same developmental stage (ripening stage). The ‘Zijing’ leaves were provided by Beijing international walnut manor in Qingshui town, Mentougou district, Beijing. The total RNA was collected using the PLANT RNA kit (50) developed by OMEGA, Norcross, GA, USA. The eligibility of RNA samples was measured with Nano drop 2000 spectrophotometer. Subsequent qRT-PCR experiments were conducted to validate the significant differences expressed by *JrR2R3-MYBs*. The walnut β-actin gene was employed as an endogenous gene [57]. Primers were devised through Primer3Plus (Table S1) [58]. qRT-PCR results were calculated using the 2^-ΔΔCT^ method [59].

### 2.7. Protein–Protein Interactions and MicroRNA Targeting Analysis

The nine JrR2R3-MYB sequences associated with walnut color regulation were input into STRING [60] to predict the interactions of these proteins. The nucleotide sequences of these 9 *JrR2R3-MYBs* were submitted to analysis using psRNATarget [61] to predict the targeted miRNAs. Visualization was conducted using Cytoscape software [62].

## 3. Results

### 3.1. Genome-Wide Identification and Chromosomal Distribution of JrR2R3-MYBs

We identified 224 *JrMYBs* according to the walnut reference genome Chandler v2.0. All identified JrMYB proteins contain the MYB domain repeat SANT, and four subfamilies were identified, including 11 *1R-JrMYBs*, 204 *JrR2R3-MYBs*, 8 *R1JrR2R3-MYBs*, and 1 *5R-JrMYB* (Figure S2). Among them, *JrR2R3-MYB* was the largest *MYB* subfamily, comprising 91.1% of the *JrMYB* gene family. To aid the subsequent study, we renamed all the *JrR2R3-MYBs* according to chromosomal position.

**Figure 1:**
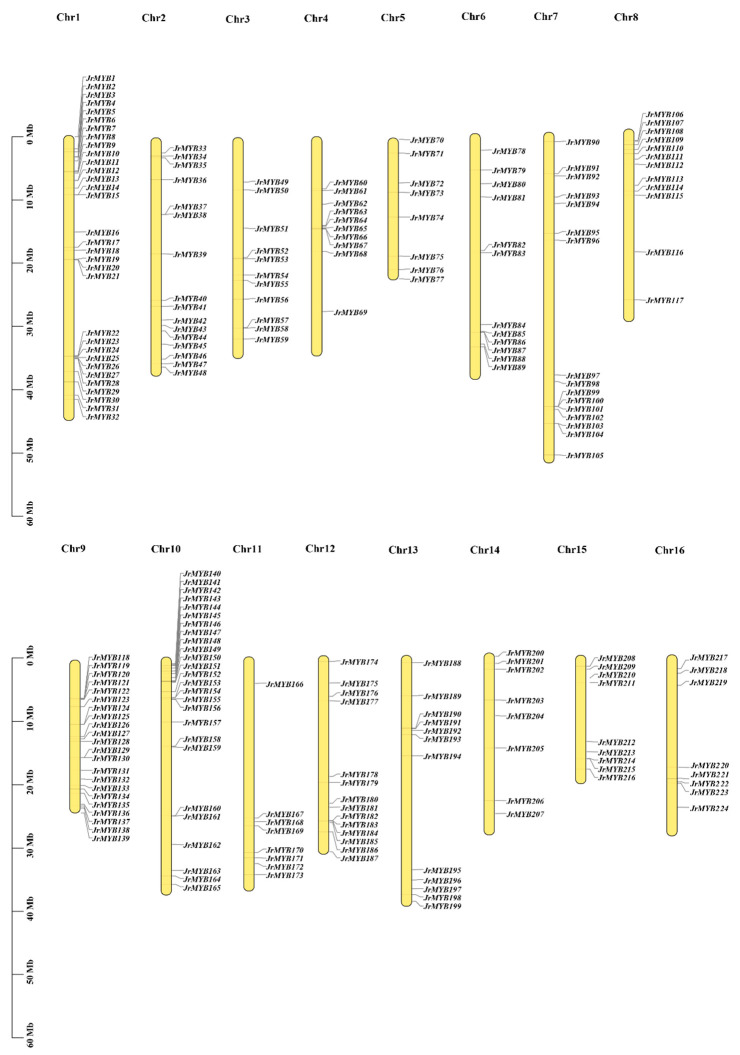
Chromosomal distribution of *JrMYB* genes.

The chromosomal locations showed that all the *JrMYBs* mapped to walnut chromosomes 1 to 16, for which 204 genes were *R2R3-MYB*. Although all 16 walnut chromosomes included some *JrR2R3-MYBs*, the allocation seemed to be non-uniform. The greatest quantity of *JrR2R3-MYBs* were found on chromosome 1, with 32 genes, while the lowest quantities were found on chromosomes 5, 11, 14, and 16, with 8 genes. The 224 *JrMYBs* were named *JrMYB1-JrMYB224* according to their locations on the 16 chromosomes (Figure 1). The density of *JrR2R3-MYBs* was relatively high in certain chromosomal regions, for example, the ends of chromosomes 1, 9, and 10 and the central section of chromosome 4. In contrast, several large chromosomal central regions lacked *JrR2R3-MYBs*, for instance, chromosomes 7, 11, 12, 13, 15, and 16.

### 3.2. Phylogenetic Analysis of JrR2R3-MYBs

**Figure 2:**
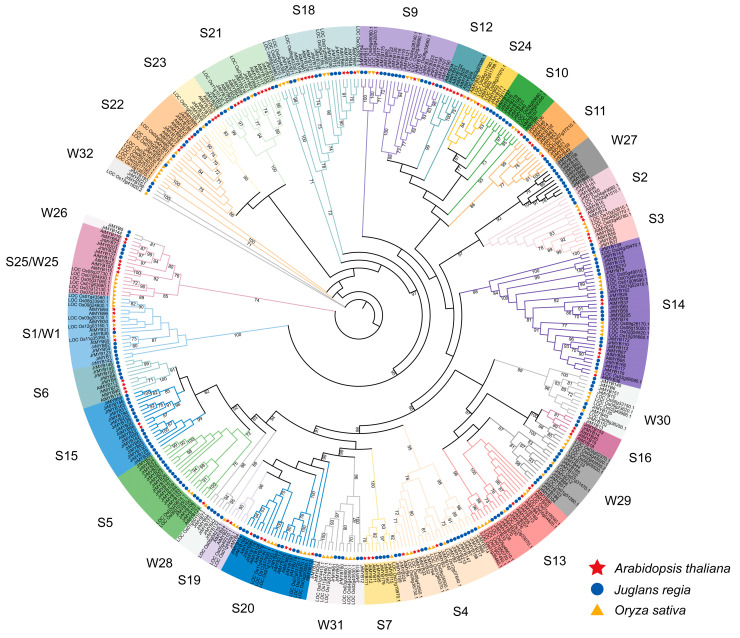
Phylogenetic relationships of R2R3-MYB proteins between *J. regia*, *O.sativa*, and *Arabidopsis*.

An ML tree containing 204 *JrR2R3-MYBs*, 90 *AtR2R3-MYBs*, and 99 *OsR2R3-MYBs* was constructed to analyze the phylogenetic relationships (Figure 2). All the members of the *JrR2R3-MYB* family can be divided into 30 subgroups (W1-W32 without W8 and W17) based on the results of the phylogenetic analysis, among which groups W1-W25 correspond to S1-S25 in *AtMYB* of *Arabidopsis*. Most of them contain *R2R3-MYBs* from three species at the same time, indicating a close phylogenetic relationship between them. Notably, there were seven subgroups (W26-W32) that were clustered only with *JrR2R3-MYBs* and *OsR2R3-MYBs*, suggesting that these genes may have evolved independently of each other after the divergence of walnuts or rice. In addition, the results based on branch-length variations showed that individual gene pairs had longer evolutionary branches between them (Figure S3), suggesting that these genes may have undergone large mutations during evolution (*Os05g37730* and *Os01g04930*; and *JrMYB156*, *JrMYB211,* and *JrMYB193*). Previous studies have shown that four branches, S4, S5, S6, and S7, are involved in the plant flavonoid metabolic pathway and anthocyanin synthesis. The S4 subgroup encodes transcription repressors, the S5 subgroup regulates the synthesis of proanthocyanidins in *Arabidopsis*, the S6 subgroup closely participates in anthocyanin synthesis in plant nutrient tissues, and the S7 subgroup can regulate the synthesis of flavonols. The phylogenetic relationships showed that there were 39 *JrR2R3-MYB* genes closely related to the evolution of the S4-S7 subgroups of R2R3-AtMYB.

### 3.3. Collinearity Analysis of JrR2R3-MYBs

**Figure 3:**
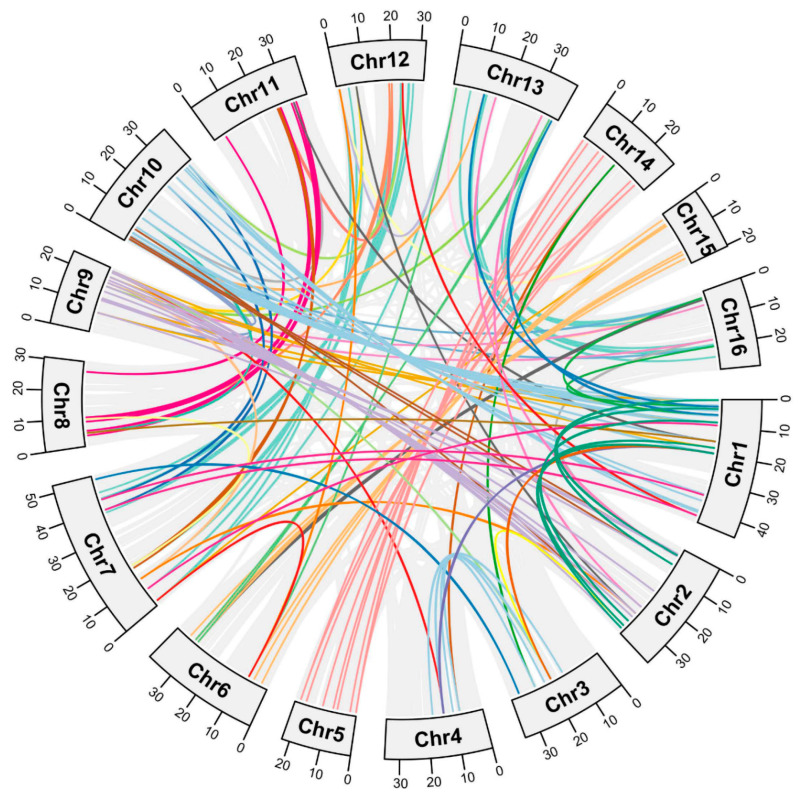
Collinearity analyses of *JrR2R3-MYB* genes. Different color lines indicate paralogous gene pairs.

This study investigated gene duplication events, including whole-genome duplication (WGD) or segmental duplication, proximal duplication (PD), and tandem duplication (TD), and aims to elucidate the expansion mechanism of *JrR2R3-MYBs* developed during evolution. We found that WGD duplication accounted for 158 of the 204 *JrR2R3-MYBs* (77.45%). There were 21 *JrR2R3-MYBs* that underwent TD (10.30%), while 17 *JrR2R3-MYBs* experienced DSD (8.33%), and 8 *JrR2R3-MYBs* experienced PD (3.92%, Figure S4; Table S2). Walnut contains subgenomes, which were divided into two groups of homologous subgenomes, namely, a dominant subgenome (DS) and a submissive subgenome (SS), and the 16 chromosomes of walnut were divided into eight pairs of chromosomes based on their homologous relationships [63]. Among them, Chr1 and Chr10, Chr2 and Chr9, Chr3 and Chr4, Chr6 and Chr15, Chr7 and Chr12, Chr11 and Chr8, Chr13 and Chr16, and Chr14 and Chr5 are homologous chromosomes with respect to each other. There were 98 *JrR2R3-MYBs* (48.04%) that have homologous counterparts in the syntenic region of related chromosomes (Table S3). In addition, 148 homologous *JrR2R3-MYB* gene pairs were identified (Figure 3). Based on synonymous (Ks) and non-synonymous (Ka) values, it was determined that 148 homologous gene pairs had Ka/Ks ratios less than 1, demonstrating that these genes are under negative selection (Table S3).

**Figure 4:**
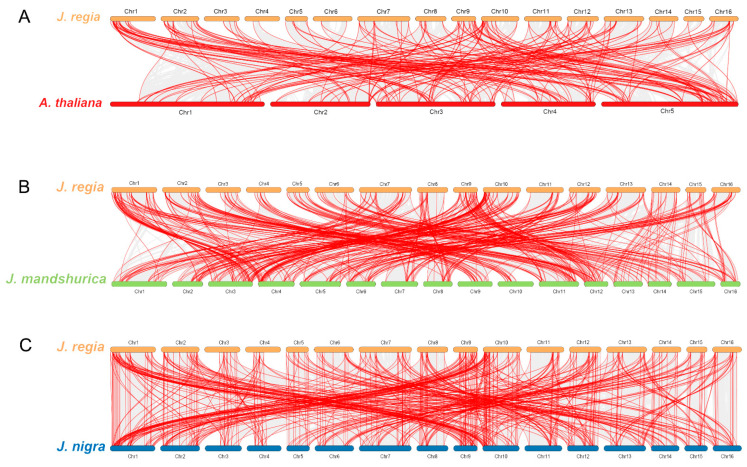
The collinearity relationships of *R2R3-MYB* genes. (**A**) Collinearity relationships of *R2R3-MYB* genes among *J. regia* and *Arabidopsis*; (**B**) collinearity relationships of *R2R3-MYB* genes among *J. regia* and *J. mandshurica*; (**C**) collinearity relationships of *R2R3-MYB* genes among *J. regia* and *J. nigra*. Grey lines indicate orthologous gene pairs and red lines indicate orthologous *R2R3-MYB* gene pairs.

Furthermore, to inspect the potential evolution of *R2R3-MYBs* of the common walnut, we performed a collinearity analysis between three *Juglans* species (*J. regia*, *J. mandshurica,* and *J. nigra*) and *Arabidopsis*. Walnut and Arabidopsis have 208 *JrR2R3-MYB* homologous gene pairs (Figure 4A). *JrR2R3-MYBs* have 434 homologous gene pairs with respect to *J. mandshurica* (Figure 4B) and 492 homologous gene pairs with respect to *J. nigra* (Figure 4C). These results indicate that the three *Juglans* species are more tightly involved with each other than *Arabidopsis*. In comparison, walnut was more closely related to *J. nigra* than to *J. mandshurica*.

### 3.4. Physicochemical Properties and Prediction of Subcellular Locations

The 204 JrR2R3-MYB proteins ranged in length from 118 aa (JrMYB107) to 1009 aa (JrMYB156); the average length was 328 aa. The molecular weight of all JrR2R3-MYBs ranged from 13.5 kDa (JrMYB107) to 113.0 kDa (JrMYB156); the average molecular weight was 36.9 kDa. There were 126 acidic proteins (with an isoelectric point < 7) and 78 basic proteins (with an isoelectric point > 7), with an average isoelectric point of 6.91. Among these proteins, there were 13 with instability index values less than 40, while the others had values greater than 40, indicating that there were only 13 stable proteins. In addition, the GRAVY (grand average of hydropathicity) of all the JrR2R3-MYBs in *J. regia* was negative, showing that JrR2R3-MYBs are hydrophilic. As expected, all the *JrR2R3-MYBs* were found to be situated in the nucleus (Table S4).

### 3.5. Characteristics of JrR2R3-MYBs

**Figure 5:**
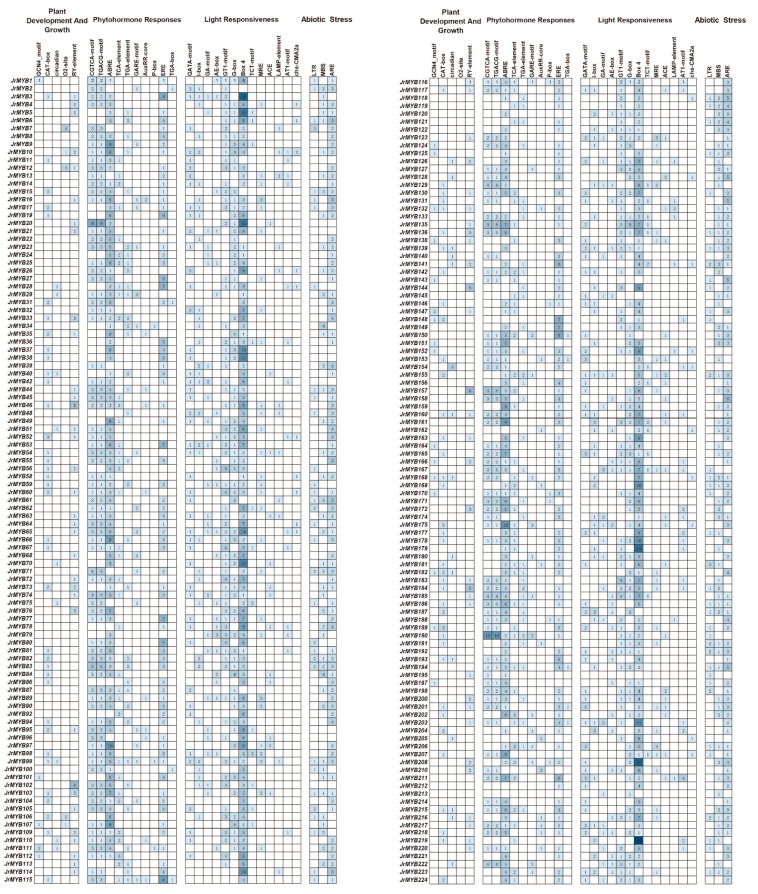
The analysis of all JrR2R3-MYB *cis*-acting elements. The colored numbers indicate the number of *cis*-acting elements.

The prediction of *cis*-acting elements revealed four major functional categories: plant growth and development, light response, metabolic response, and stress response (Figure 5 and Figure S5). The highest number of light-responsive elements was 1068, followed by 621 gibberellic acid-responsive elements and 524 MeJA-responsive elements. Furthermore, elements related to flavonoid regulation were predicted, indicating that JrR2R3-MYB might be closely associated with the flavonoid metabolic pathway.

We detected 20 conservative motifs from among all the *JrR2R3-MYB* members. The number of amino acids per conserved motif varies from 8 to 50. All *JrR2R3-MYBs* contained motif 2 and motif 3, whereas motif 9, motif 11, motif 14, and motif 18 only existed in a few members. Generally, the same subpopulation has not only comparable features but also similar motifs (Figure S6).

Gene architecture analysis revealed that the exon numbers of *JrR2R3-MYBs* varied from 1 to 12, with an average of 3. The range of exon numbers varied greatly, but the majority of the structures of the *JrR2R3-MYB* genes still consisted of three exons. The results suggest that genes on the identical branches might have comparable exon–intron structures (Figure S7).

### 3.6. Expression Profiles of JrR2R3-MYBs

**Figure 6:**
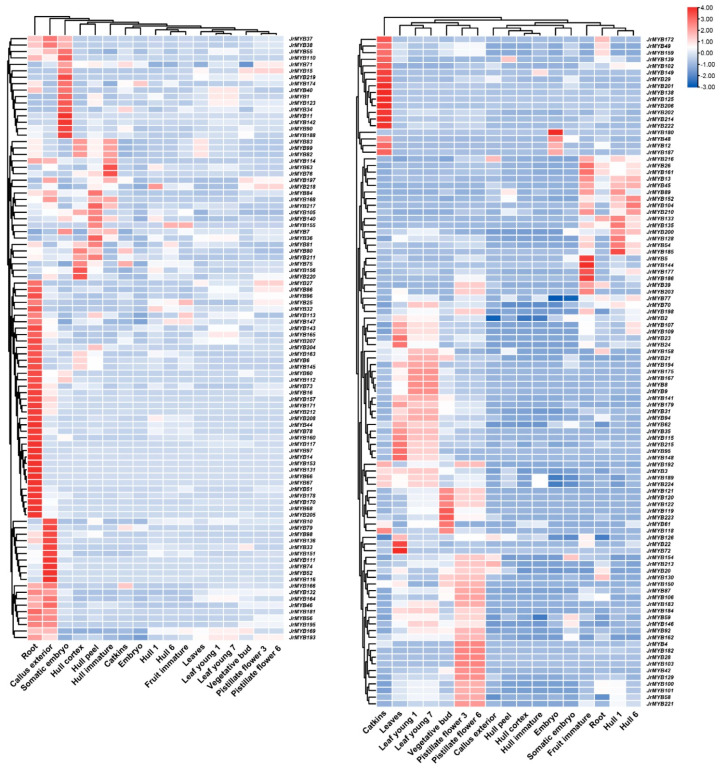
Gene expression levels of *JrR2R3-MYBs* in different organs.

To explore the expression patterns of *JrR2R3-MYBs*, we visualized all the *R2R3-MYB* genes identified based on transcriptomic data present in vegetative buds, embryos, somatic embryos, young leaves, leaves, roots, callus exterior, pistillate flowers, catkins, hull peels, hull cortexes, immature hulls, hulls, and immature fruit expressed in 14 selected organs (Figure 6, Table S5). The transcriptome results showed that all 196 *JrR2R3-MYB* genes except *JrMYB17*, *JrMYB19*, *JrMYB53*, *JrMYB64*, *JrMYB65*, *JrMYB124*, *JrMYB190*, and *JrMYB191* were expressed in the selected tissues. The eight unexpressed genes may be expressed in other developing organs or during other developmental periods. Most of the *JrR2R3-MYBs* were highly expressed in the roots, leaves, catkins, pistillate flowers, and callus exterior. The different expression patterns in different organs suggested that *JrR2R3-MYB* genes play different roles in the growth and development of walnuts.

**Figure 7:**
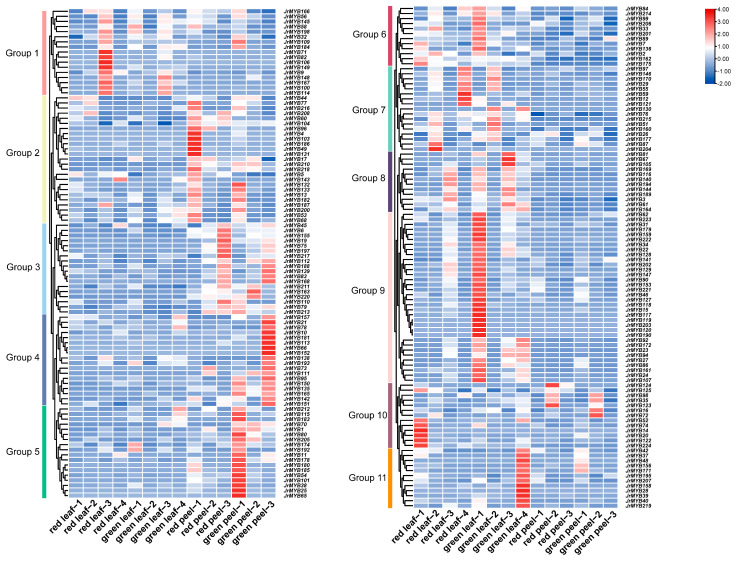
Gene expression patterns of *JrR2R3-MYBs* in different organs in red walnut and green walnut.

Subsequently, to investigate the regulation of walnut color by *JrR2R3-MYB* genes, the expression profiles of all the identified *R2R3-MYBs* were analyzed in the leaves and peels of red and green walnuts at various stations of development (Figure 7, Table S6). These genes were classified into 11 groups according to their expression patterns. The *JrR2R3-MYBs* in Group 1 had higher expression in red walnut leaves at the fruit-swelling stage (red-leaf_3). The *JrR2R3-MYBs* in Group 2 were highly expressed in early peels and expressed at much higher levels in red peels than in green peels. The *JrR2R3-MYBs* in Groups 4, 5, 9, and 11 were highly expressed only in green walnut leaves or peels, demonstrating that these genes may participate in the regulation of walnut color regulation. The *JrR2R3-MYBs* in Group 3 showed similar expression profiles in peels at various stages of development in red and green walnut varieties.

### 3.7. Identification of Differentially Expressed R2R3-MYBs Related to Coloration in the ‘Zijing’ Walnut

**Figure 8:**
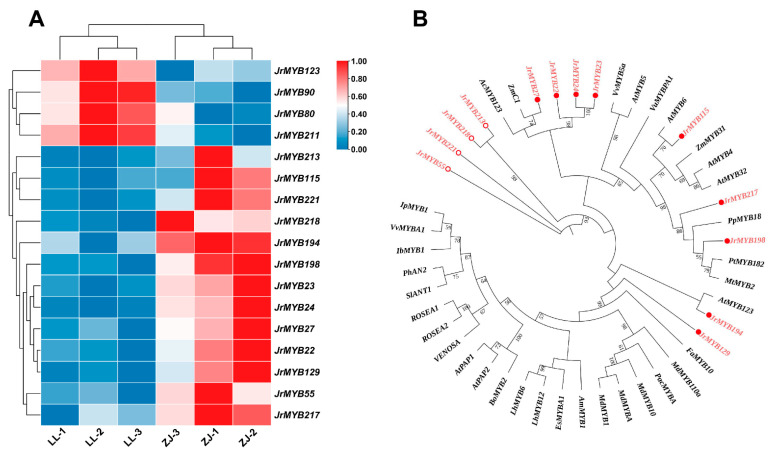
The DEGs in ‘Zijing’ and ‘Lvling’ walnut varieties. (**A**) A heat map of 17 differentially expressed *JrR2R3-MYBs* in differently colored leaves of two walnut varieties ‘Zijing’ and ‘Lvling’ obtained using RNA-seq. (**B**) Phylogenetic analysis of the discrepancy-expressed *JrR2R3-MYBs* in ‘Zijing’ walnut and anthocyanin-related *R2R3-MYBs.* Red fronts represent JrR2R3-MYBs, and circles represent *JrR2R3-MYBs* in ‘Zijing’ walnut.

The leaves of ‘Zijing’ and ‘Lvling’ walnut plants with different colors at the same developmental stage were used as materials for transcriptome sequencing. After removing low-quality reads, a complete set of 42.20 Gb of clean data was obtained for the six specimens, with an average of 6.27 Gb per sample. The clean data were mapped to the *J. reiga* reference genome Chandler v2.0, with alignment ratios ranging from 94.06% to 94.84%. It was found that 17 *JrR2R3-MYBs* were discrepancy-expressed in ‘Zijing’ and ‘Lvling’ leaves, among which 13 DEGs were expressed to a greater degree in ‘Zijing’ than in ‘Lvling’ (Figure 8A).

An ML tree was constructed using the 13 *JrR2R3-MYB* DEGs and additional reported *R2R3-MYBs* associated with anthocyanin synthesis in various species (Figure 8B). We found nine genes, namely, *JrMYB22*, *JrMYB23*, *JrMYB24, JrMYB27*, *JrMYB115*, *JrMYB129, JrMYB194*, *JrMYB198*, and *JrMYB217*, that might regulate the anthocyanin synthesis of walnut’s purplish-red leaves. As shown in Figure 6, *JrMYB22*, *JrMYB23*, *JrMYB24,* and *JrMYB27* were close homologs with respect to *ZmC1*, demonstrating that they might positively regulate anthocyanin synthesis [64], and *JrMYB194* was observed to be a close homolog with respect to *AtMYB123*, demonstrating that it possibly induces the activity of the late-biosynthesis genes (LBGs) for anthocyanins and proanthocyanins [34]. *AtMYB4* and *ZmMYB31* share similar evolutionary relationships with *JrMYB115*, *JrMYB217*, and *JrMYB198*, which were determined to be able to modulate the accumulation of the UV-protectant compound sinapoylmalate via transcriptional inhibition of the gene coding for the phenylpropanoid enzyme cinnamate 4-hydroxylase or directly repress maize lignin genes and alteration in the direction of phenylpropanoid metabolic fluxes [65,66]. *JrMYB129* was clustered in the same clade with the well-known transcription factors *MdMYB10*, *MdMYB1*, and *FaMYB1* that induce anthocyanin synthesis, suggesting that it possibly participates in controlling anthocyanin synthesis [67,68,69].

**Figure 9:**
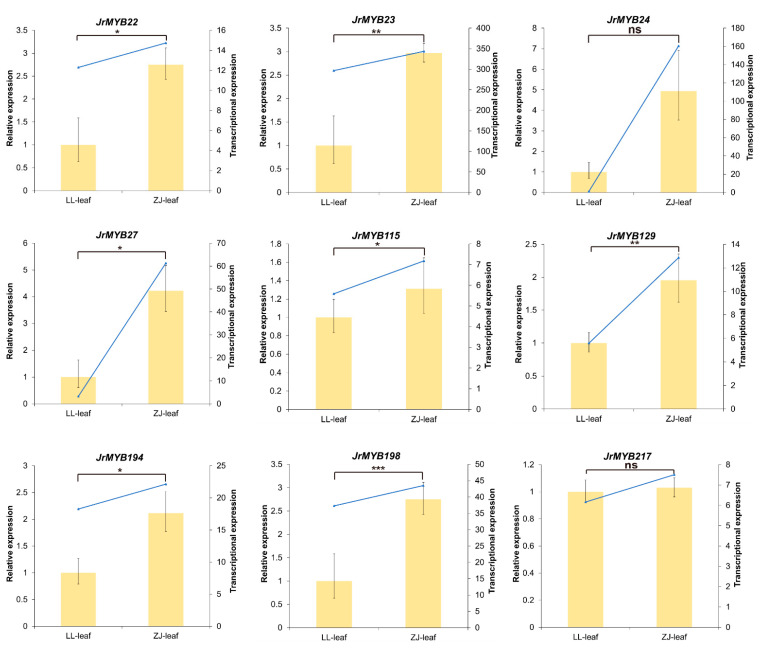
Relative expression of 9 *JrR2R3-MYBs* in ‘Zijing’ and ‘Lvling’ leaves. LL represents ‘Lvling’, ZJ represents ‘Zijing’. The yellow bars represent qRT-PCR results, while the bule lines represent FPKM value. ns = no significant different, * = *p* < 0.05, ** = *p* < 0.01, and *** = *p* < 0.001.

To probe the relative expression levels of the nine *JrR2R3-MYBs* above in the two walnut varieties, we performed qRT-PCR experiments. The results revealed that the expression of these nine *JrR2R3-MYBs* in purplish-red walnuts was higher than that in green walnuts (Figure 9). These results further indicated that these nine *JrR2R3-MYBs* might be involved in controlling anthocyanins in the purplish-red leaves of ‘Zijing’ walnut.

**Figure 10:**
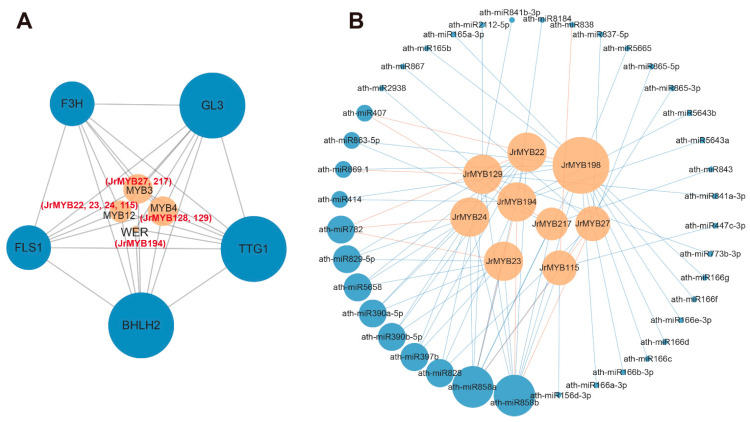
Network analysis of *JrR2R3-MYB* members. (**A**) Protein–protein interactions of the nine JrR2R3-MYB proteins related to ‘Zijing’ walnut color regulation JrR2R3-MYB proteins. Red font indicates JrR2R3-MYB proteins. (**B**) MiRNA targeting of the nine *JrR2R3-MYB* genes related to ‘Zijing’ walnut color regulation. The blue circle and orange circle represent miRNAs and *JrR2R3-MYBs*, respectively. Blue lines represent cleavage, and orange lines represent translation.

We predicted the interactions of nine JrR2R3-MYB proteins related to ‘Zijing’ walnut color regulation according to the interactions of MYB proteins in *Arabidopsis thaliana* using homology profiling (Figure 10A). The interactions between the nine JrR2R3-MYB proteins investigated and the proteins bHLH2, TTG1, GL3, F3H, and FLS1 suggest that the mentioned genes co-regulate the anthocyanin synthesis pathway. A total of 1274 microRNAs were predicted to target 201 *JrR2R3-MYB* genes (Table S6), of which 74 microRNAs targeted 9 *JrR2R3-MYB* genes associated with ‘Zijing’ walnut color regulation (Figure 10B). Furthermore, 16 miRNAs of these 74 miRNAs regulated gene expression through cleavage, and 14 miRNAs regulated gene expression through translation, suggesting that cleavage is the main way miRNAs regulate *JrR2R3-MYB* genes.

## 4. Discussion

### 4.1. Characterization of the JrR2R3-MYBs

The *MYB* gene family is among the greatest gene families in plants, and a wealth of evidence shows that it could be implicated in a wide range of plant metabolic pathways [70]. As the greatest subfamily of the *MYB* family, the *R2R3-MYB* subfamily is responsible for most of the functions of the *MYB* family, including regulating plant flavonoid metabolism and anthocyanin synthesis [71,72]. Walnut is a significant resource species worldwide; their fruit ranks first among the world’s four largest nuts [73], and other parts such as branches, pollen, and husk also have potential value. These gene family members derive from the same ancestral gene, have comparable structures and functions, and encode similar proteins, but most of them have different expression regulation patterns and different functions [74]. The study of gene families can not only delineate the evolutionary history of genes but also quickly identify members related to target traits in target species and make the study of gene molecular biological functions more convenient. As Chandler v2.0 was published with a new chromosome-level assembly, we have obtained more precise reference genome data to explore many still-unanswered questions regarding walnuts [75]. To date, *JrR2R3-MYBs* have yet to be comprehensively analyzed, and the majority of the functions of *R2R3-MYB* genes remain unknown. In this study, we detected 204 *JrR2R3-MYB* genes from the Chandler v2.0 genome (Figure 1), and the major nuclear localization of these genes is compatible with their roles as transcription factors (Table S3). R2R3-MYB is a large family, with 55 members that can be divided into 11 subgroups in *Cucumis sativus* [76], 184 members that can be divided into 34 subgroups in pear [77], and 100 members that can be divided into 29 subgroups in *Citrus sinensis* [78]. In comparison, *R2R3-MYB* members are more numerous in walnut. The phylogenetic analysis determined that *JrR2R3-MYB* was clustered into 30 subgroups (Figure 2, S3). Notably, no homolog genes of *JrMYB* were found in group S12 according to the *Arabidopsis* subgroup classification. Comparable results were found in octoploid strawberry [79], which lacks members in subgroup S12 that were possibly lost during evolution. This study provides novel inspirations for future investigators seeking to determine functional distinctions in *JrR2R3-MYBs*.

### 4.2. Gene Duplication and Evolution of JrR2R3-MYBs

Gene family expansion and the creation of new genes arise from gene duplication events [80,81]. *JrR2R3-MYBs* were found on every chromosome, but they were unequally spread out (Figure 3). There were a lot of gene duplication events in the *JrR2R3-MYBs.* According to our statistics, 98 *JrR2R3-MYBs* (48.04%) have homologous counterparts in the syntenic region of related chromosomes (Table S3). In a comparative analysis of R2R3-MYBs in land plants, more than 20% of *R2R3-MYBs* in each species were found to have homologous counterparts in the syntenic region of related chromosomes [82], with 49% in *SlR2R3-MYBs*, 48% in *AtR2R3-MYBs*, 38% in *PtR2R3-MYBs*, and 38% in *VvR2R3-MYBs*. This finding is similar to the results for *JrR2R3-MYBs*. These results suggest that WGD events are the main reason for the expansion of the *JrR2R3-MYB* gene family (Figure S3; Table S2). In contrast, WGD and TD events promote *R2R3-MYB* extension in *M. truncatula* [83], and all duplication events observed in sweet orange were segmental duplications [80]. Compared with the high level of collinearity of *R2R3-MYB* genes in walnuts, the duplication events in genes of these species appear to be quite limited, which may be part of the reason why there were fewer members than in walnuts. There were high degrees of collinearity in the *R2R3-MYBs* in the three *Juglans* species, and walnut is more closely related to *J. nigra* than to *J. mandshurica* (Figure 4).

### 4.3. Functional Prediction of JrR2R3-MYBs in ‘Zijing’ Walnut

Most of the identified *JrR2R3-MYBs* were expressed in the 14 selected organs, suggesting that *R2R3-MYBs* extensively participate in the growth and development of walnuts (Figure 6). Special cultivars such as ‘Hongrang’, ‘Hongren’, ‘Ziyue’ (*Juglans sigillata*), and ‘Zijing’ increased the ornamental and economical value of walnut. Some of the *JrR2R3-MYBs* were highly expressed only in red or green walnuts, suggesting this family’s involvement in the process of walnut color regulation (Figure 7).

The ‘Zijing’ walnut, which originated in Beijing, China, can be used in landscaping or landscape agriculture because of its majestic crown, luxuriant branches, and bright leaves [6]. Compared with the common green-fruit walnuts, all the organs of the ‘Zijing’ walnut are purplish red, provoking people to pay more attention to walnut anthocyanin metabolism. However, not much is known about the genes implicated in walnut coloration. We identified putative *JrR2R3-MYBs* via RNA-seq using different leaf colors of the two walnut cultivars. In this study, 17 *JrR2R3-MYBs* were found, and 13 of them were highly expressed in ‘Zijing’, while 4 were highly expressed in ‘Lvling’ (Figure 8). We constructed an ML tree including 13 *JrR2R3-MYBs* and 34 genes from other species that were known to regulate anthocyanins to explore whether *JrR2R3-MYBs* can affect the synthesis of anthocyanins in walnut leaves; finally, 9 genes were screened. *AcMYB123* and *ZmC1* share high homology with *JrMYB22*, *JrMYB23*, *JrMYB24*, and *JrMYB27*, which could induce anthocyanin biosynthesis in kiwifruit and maize [64,84]. *JrMYB115*, *JrMYB194*, and *JrMYB129* share a similar evolutionary relationship with many well-known genes involved in anthocyanin synthesis, such as *AtMYB6*, *FaMYB10*, *MdMYB1*, and *AtMYB123* [34,85,86]. Unexpectedly, *MtMYB2* is a transcriptional repressor that regulates anthocyanin and PA biosynthesis in *M. truncatula* [87], and it has a very close evolutionary relationship with *JrMYB217* and *JrMYB198*. We performed qRT-PCR using the purplish-red leaves of the ‘Zijing’ walnut and the green leaves of the ‘Lvling’ walnut as plant materials; it was found that nine *JrR2R3-MYB* genes showed high expression in purplish-red leaves (Figure 9). These results indicate that these nine *JrR2R3-MYBs* should be regarded as significant candidate genes participating in anthocyanin biosynthesis regulation. These data revealed the possibility of influencing anthocyanin biosynthesis in the husk of the ‘Zijing’ walnut, thereby affecting walnut coloration.

However, *JrR2R3-MYB* has not been previously reported to regulate the expression levels of structural genes in the flavonoid metabolism pathway in walnuts by interacting with *bHLH* and *WD40.* Our final identification of nine *JrR2R3-MYBs* suggested that they perform a crucial role in the color formation of ‘Zijing‘ walnut leaves and may interact with *bHLH*, *TTG*, *GL*, *F3H*, and *FLS* (Figure 10), but whether they interact with other structural genes to play a part in anthocyanin synthesis is unknown for these reasons. The specific mechanisms need to be studied in a more in-depth manner.

## 5. Conclusions

This study proceeded as follows: we performed a detailed genome-wide analysis of *JrR2R3-MYBs* in walnuts, and 224 *JrMYBs* were determined and renamed based on their chromosomal locations. Among the 204 *JrR2R3-MYB* genes, the protein physicochemical properties, subcellular location, phylogenetic relationship, cis-elements, gene structure, conservative motifs, and gene replication events were studied. All *JrR2R3-MYB* genes are unevenly distributed on 16 chromosomes. According to their phylogenetic relationships, they can be divided into 30 subgroups. Collinearity analysis showed that the expansion of *JrR2R3-MYB* genes is related to WGD events. Through the screening of differentially expressed genes in the transcriptome combined with qRT-PCR verification, we identified nine *JrR2R3-MYBs* that may participate in the synthesis of anthocyanins in the purplish-red husks of walnuts. These results offer a rationale for the identification of *R2R3-MYBs* that affect anthocyanins in plants and set the stage for the further exploration of the functional characteristics of *JrR2R3-MYBs*.

## Data Availability

The raw data were downloaded from the SRA database under accession numbers GSE162007 and PRJNA688391.

## Supplementary Materials

The following are available online at https://www.mdpi.com/article/10.3390/genes15050587/s1. Figure S1: Morphological picture of ‘Zijing’ and ‘Lvling’ walnut varieties; Figure S2: Conserved domains of 224 *JrMYB* in *J. regia*., with a total of 204 JrR2R3-MYB proteins containing 2 SANT domains; Figure S3: Phylogenetic relationships of R2R3-MYB proteins between *J. regia, O. sativa,* and *Arabidopsis* display branch length variation; Figure S4: The 204 *JrR2R3-MYB* member duplicates in *J. regia*; Figure S5: *Cis*-element analysis of the *JrR2R3-MYB* gene family; Figure S6: Location of conservative motifs in *JrR2R3-MYB* and their systematic evolutionary relationship; Figure S7: The gene structures and phylogenetic relationships of *JrR2R3-MYB*. The number of exons ranges from 1–12, and the genes with 3 exons account for the majority; Table S1: The primers for qRT-PCR of *JrR2R3-MYBs*; Table S2: The four duplicated types of *JrR2R3-MYBs*; Table S3: Estimated Ka/Ks ratios of duplicated *JrR2R3-MYB* gene pairs; Table S4: The predicted protein information of JrR2R3-MYBs; Table S5: The FPKM of all *JrR2R3-MYB* genes in 14 issues; Table S6: The FPKM value of all *JrR2R3-MYB* genes in different walnuts; Table S7: Predicted miRNAs targeting *JrR2R3-MYB* genes.
