# Supplementary figures and images for: Genome-Wide Analysis of Transcription Factor R2R3-MYB Gene Family and Gene Expression Profiles during Anthocyanin Synthesis in Common Walnut (Juglans regia L.)
Source: Genes (Basel). 2024 May 5;15(5):587. doi: 10.3390/genes15050587 (PMC11121633; doi:10.3390/genes15050587)

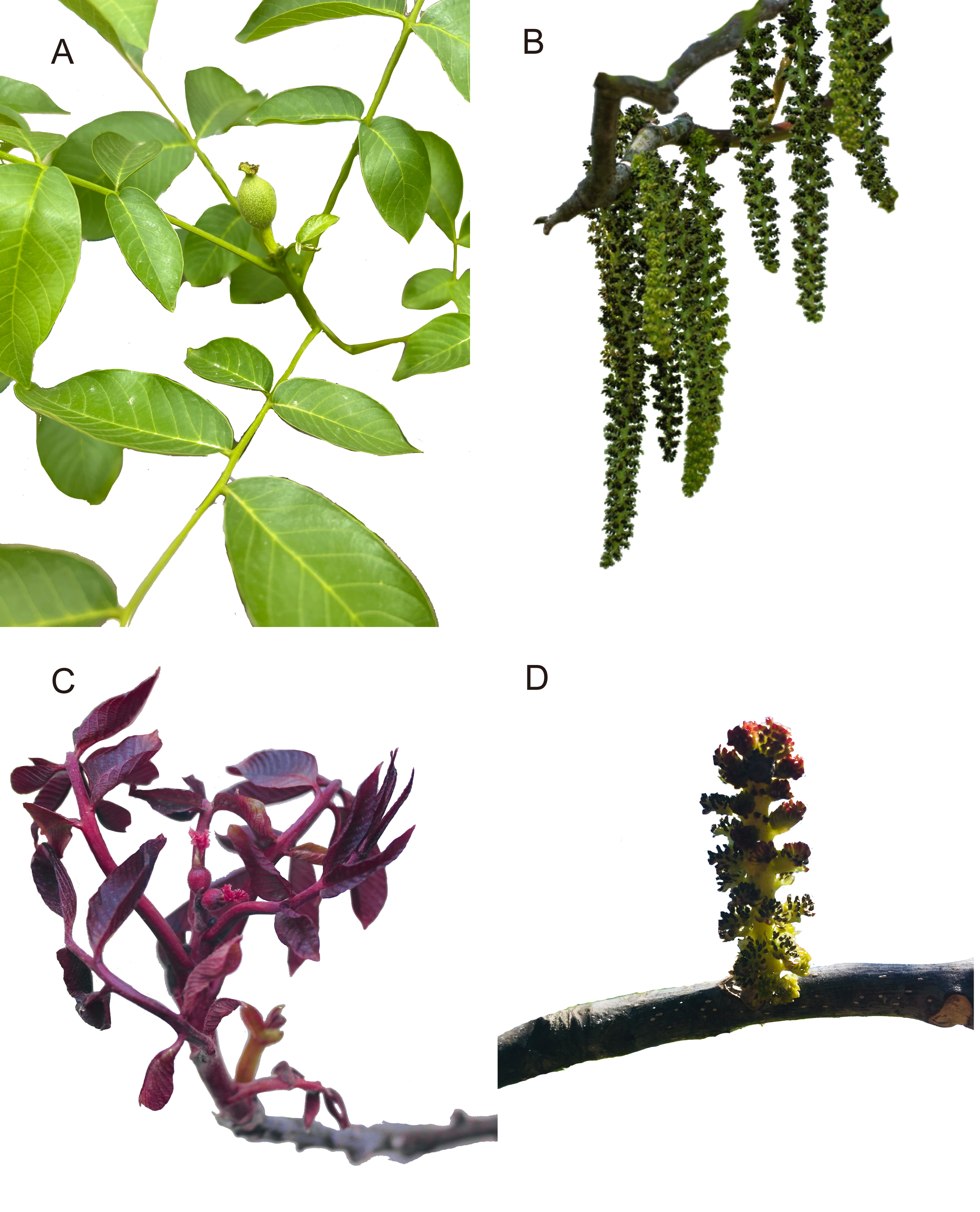

Supplement: Supplementary file 1 [file genes-15-00587-s001.zip › Figure S1.tif]

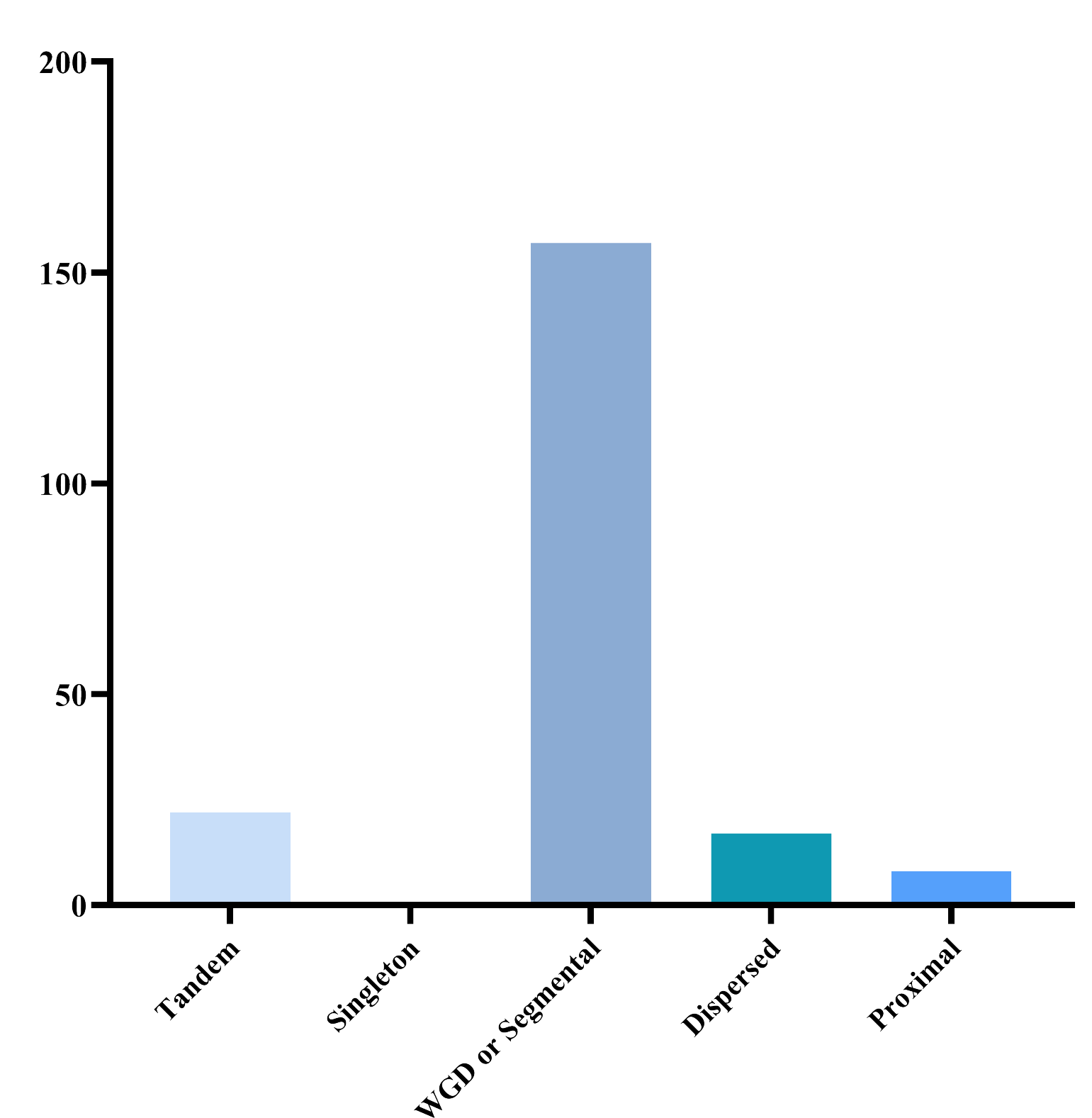

Supplement: Supplementary file 1 [file genes-15-00587-s001.zip › Figure S4.tif]

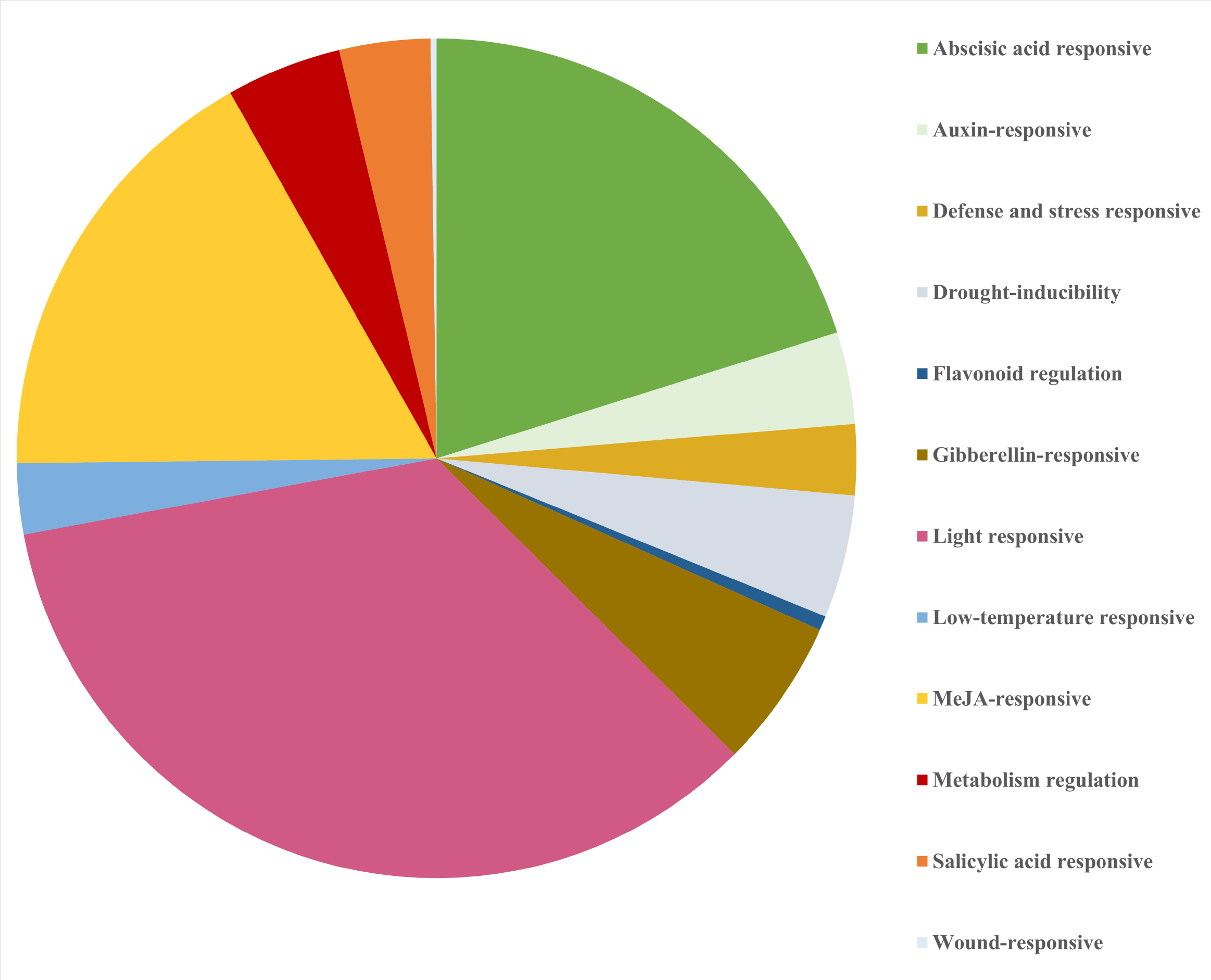

Supplement: Supplementary file 1 [file genes-15-00587-s001.zip › Figure S5.tif]
